# Identification of novel immune-related signatures for keloid diagnosis and treatment: insights from integrated bulk RNA-seq and scRNA-seq analysis

**DOI:** 10.1186/s40246-024-00647-z

**Published:** 2024-07-16

**Authors:** Kui Xiao, Sisi Wang, Wenxin Chen, Yiping Hu, Ziang Chen, Peng Liu, Jinli Zhang, Bin Chen, Zhi Zhang, Xiaojian Li

**Affiliations:** 1 Department of Plastic Surgery, Guangzhou Red Cross Hospital, Jinan University, Guangzhou, China; 2 Department of Gynaecology and Obstetrics, Hengyang Central Hospital, Hunan Normal University, Hengyang, China

**Keywords:** Keloid, Immune genes, Machine learning, Artificial neural network, ScRNA-seq, Fibroblast, Retinoic acid

## Abstract

**Background:**

Keloid is a disease characterized by proliferation of fibrous tissue after the healing of skin tissue, which seriously affects the daily life of patients. However, the clinical treatment of keloids still has limitations, that is, it is not effective in controlling keloids, resulting in a high recurrence rate. Thus, it is urgent to identify new signatures to improve the diagnosis and treatment of keloids.

**Method:**

Bulk RNA seq and scRNA seq data were downloaded from the GEO database. First, we used WGCNA and MEGENA to co-identify keloid/immune-related DEGs. Subsequently, we used three machine learning algorithms (Randomforest, SVM-RFE, and LASSO) to identify hub immune-related genes of keloid (KHIGs) and investigated the heterogeneous expression of KHIGs during fibroblast subpopulation differentiation using scRNA-seq. Finally, we used HE and Masson staining, quantitative reverse transcription-PCR, western blotting, immunohistochemical, and Immunofluorescent assay to investigate the dysregulated expression and the mechanism of retinoic acid in keloids.

**Results:**

In the present study, we identified PTGFR, RBP5, and LIF as KHIGs and validated their diagnostic performance. Subsequently, we constructed a novel artificial neural network molecular diagnostic model based on the transcriptome pattern of KHIGs, which is expected to break through the current dilemma faced by molecular diagnosis of keloids in the clinic. Meanwhile, the constructed IG score can also effectively predict keloid risk, which provides a new strategy for keloid prevention. Additionally, we observed that KHIGs were also heterogeneously expressed in the constructed differentiation trajectories of fibroblast subtypes, which may affect the differentiation of fibroblast subtypes and thus lead to dysregulation of the immune microenvironment in keloids. Finally, we found that retinoic acid may treat or alleviate keloids by inhibiting RBP5 to differentiate pro-inflammatory fibroblasts (PIF) to mesenchymal fibroblasts (MF), which further reduces collagen secretion.

**Conclusion:**

In summary, the present study provides novel immune signatures (PTGFR, RBP5, and LIF) for keloid diagnosis and treatment, and identifies retinoic acid as potential anti-keloid drugs. More importantly, we provide a new perspective for understanding the interactions between different fibroblast subtypes in keloids and the remodeling of their immune microenvironment.

**Supplementary Information:**

The online version contains supplementary material available at 10.1186/s40246-024-00647-z.

## Introduction

Keloid is mainly caused by traumatic or inflammatory skin damage, and it is widely believed that keloid formation is based on dysfunctional tissue repair [1]. However, keloids are not scars in the usual sense because they extend far beyond the original lesion area [2]. The most common pier sites are the chest, shoulder, back, and earlobes [3]. There are numerous treatment methods for keloids, including surgical and non-surgical approaches and comprehensive treatment that combines various methods. However, it has not been effectively controlled, and the recurrence rate remains very high [4]. Surgical resection and postoperative radiotherapy are still the primary methods for treating keloid lesions, but the recurrence rate after postoperative radiotherapy can also reach 16% [5].

Studies have shown that the cause of keloids is not fully understood, but fibroblasts are known to be the primary effector cells in keloid development [6]. Among these factors, TGF-β is believed to be closely associated with the increased proliferation activity of fibroblasts in keloids, enhanced invasiveness, and excessive collagen deposition [7]. Keloids are composed of a variety of subtypes of fibroblasts, which include mesenchymal fibroblast (MF), pro-inflammatory fibroblast (PIF), secretory-papillary fibroblast (SPF), secretory-reticular fibroblast (SRF) [8]. There has been increasing evidence that dysregulation of immune genes affects wound healing and leads to keloid formation [9] and that levels of various immune cells are differentially increased in keloid tissues [10]. Thus, the genome-wide revelation of dysregulated immune-related genes in this direction is expected to identify immune-related signatures that could help investigate the molecular features and mechanisms of keloid development and provide new strategies for treating keloids. Recently, high-throughput sequencing has been the hottest topic in “omics” research [11]. Machine learning is also valuable for analyzing multi-dimensional transcriptome data and identifying biologically significant genes [12, 13]. Single cell RNA sequencing is a major innovation and technological advancement in the life sciences, which provides gene expression information at the individual cell level and is essential for revealing cellular heterogeneity [14]. Thus, bulk RNA-seq combined with scRNA seq can identify different gene expression features and heterogeneity of fibroblast subtypes in keloid scars.

In summary, we performed the present study to identify and elucidate the role of immune signatures in keloids. First, we applied Weighted Gene Co-expression Network Analysis (WGCNA), Multiscale Embedded Gene Co-expression Network Analysis (MEGENA), and three machine learning algorithms to screen for hub immune-related genes of keloid. Next, we analyzed the heterogeneous expression of KHIGS in keloid fibroblast subtypes using single-cell data. Finally, we identified the promising novel drugs by targeting KHIGs, among which retinoic acid can treat keloid by reducing the differentiation of PIF to MF. This provides a new perspective for treating keloid.

## Materials and methods

### Data download and processing

Both keloid bulk RNA seq (GSE44270 and GSE7890) and scRNA seq (GSE163973) used in the present study were downloaded from the GEO database (https://www.ncbi.nlm.nih.gov/geo/), the details of which are shown in Table 1. We grouped samples derived from normal skin tissues into the normal group, while samples derived from keloid tissues were considered as the keloid group. In dataset GSE7890, we selected samples of keloid and normal skin that were not treated with hydrocortisone. In the GSE44270 dataset, we selected fibroblast samples from non-lesional, control and keloid. Additionally, since keloids usually occur under the age of 40 and in various locations [15–18]. Therefore, the age of the patients and the locations of the skin specimens needed to be clarified. Based on the above inclusion criteria, we excluded from the study samples that were older than 40 years of age and the location from which the skin specimen was taken was not clear. The basic information of the samples included in the present study is shown in Table S1 To eliminate the batch effect between datasets caused by different platforms, we used “ComBat” in the R package “sva” to eliminate the batch effect of the merged data array of GSE44270 and GSE7890. In addition, the immune-related genes used in the present study were annotated in the ImmPort database (https://www.immport.org/ resources), shown in Table S2 Finally, we used the R package “Limma” for screening differentially expressed genes (DEGs) between keloid tissue and normal skin tissue [19].

**Table 1:** The basic information regarding the dataset in the present study

Dataset Type	Dataset Source	Annotation Platform	Keloid Samples	Normal Samples
Bulk RNA-seq	GSE44270	GPL6244	9	7
	GSE7890	GPL570	5	2
scRNA seq	GSE163973	GPL24676	3	3

### Co-identification of keloid-related DEGs by WGCNA and MEGENA

WGCNA is a systems biology method for describing the correlation patterns between genes in microarray samples by applying the R package “WGCNA” to construct disease-related gene network modules [20, 21]. Firstly, a weighted adjacency matrix was generated by calculating Pearson’s Correlation among all pairs of genes. The optimal soft threshold was ten, and a hierarchical clustering tree was constructed using the degree of topological encounter as distance. The module is divided by the dynamic cutting tree method. Values of sample phenotypic characteristics were turned into visual heat maps of colors. Finally, the correlation and significance between the modules and keloid were calculated, and the values of sample phenotypic characteristics were turned into visual heat maps of colors. Finally, the correlation and significance between the modules and keloid were calculated, and the correlation heatmap was drawn to determine the modules most relevant to keloid.

MEGENA can effectively construct and analyze large-scale planar filter co-expression networks [22]. We performed MEGENA analysis using the R package “MEGENA”. Multiscale hub analysis (MHA) is used to identify highly complex network clustering features (CTA). We also used Cytoscape to visualize the genes in clusters c1_2, c1_4, c1_6, and c1_15 of MEGENA. Subsequently, we used the R function “phyper” to test the hypergeometric distribution of all MEGENA and turquoise modules in WGCNA. Finally, we filtered out the MEGENA modules with significant overlap with turquoise modules in WGCNA as keloid-related gene modules under the MEGENA algorithm. Subsequently, we intersected the turquoise module of WGCNA with the keloid-related gene modules in MEGENA, and the intersected genes obtained were used as the keloid-related DEGs in the present study.

### Functional enrichment analysis

Gene Ontology (GO) term and Kyoto Encyclopedia of Genes and Genomes (KEGG) pathway enrichment analysis of keloid/immune-related DEGs were performed using the “clusterProfiler” package [23]. P value < 0.05 is a screening criterion to investigate their biological functions, signaling pathway enrichment, and disease similarities.

### Identification of hub immune-related genes of keloid

Machine learning is widely used in the biomedical field and can efficiently and rapidly analyze biological data to accurately identify hub genes in gene expression profiles [24]. We used three machine learning algorithms to identify KHIGs in the present study, namely, last absolute shrinkage and selection operator (LASSO), random forest (RF), and support vector machine recursive feature elimination (SVM-RFE). Specifically, we screened keloid/immune-related DEGs for potential candidate genes using the LASSO algorithm in the “glmnet” package, the RF algorithm in the “randomForest” package, and the SVM-RFE algorithm in the “e1071” package [25–27]. Then, we used upset diagrams to crossover the candidate genes screened by the three algorithms mentioned above and finally identified three crossover candidate genes named KHIGs.

### Construction and verification of the ANN (Artificial neural network) model

We constructed an ANN diagnostic model based on the transcriptome level of KHIGs and the clinical characteristics of the samples (age and gender) using the R package “neuralnet”. The ANN model is able to mimic the structure and function of the brain’s neural networks to derive a set of classification rules from complex and irregular data, resulting in a highly accurate diagnostic model [28]. Specifically, we use the “caret” R package to divide the samples into a training set and a testing set in the ratio of 7:3, and the detailed sample division is shown in Table S3. This division allows us to train the model on one subset and evaluate the performance of the model on a separate subset, thus ensuring a fair assessment of the accuracy of the algorithm. Next, we utilized the R packages “neuralnet” and “neuralnettools” to construct an ANN diagnostic model, which consists of three main layers: input, hidden, and output layers, all of which work in coordination to work to achieve accurate keloid classification. During the construction of the model, the input data consisted of the expression of KHIGs and the clinical characteristics of the samples (age and gender). Additionally, the output data was composed of two nodes that we defined: “Normal” for the normal group and “Keloid” for the keloid group. Each of these nodes employed a softmax activation function to ensure the model in the configuration of the key parameters of the model, we set the first hidden layer to 8 neurons and the second hidden layer to 3 neurons, and utilized the gene weight information to produce an accurate keloid classification model. Meanwhile, we reduce the risk of overfitting and underfitting by 10-fold cross-validation. Finally, we use accuracy; precision; recall; F1-score and AUC to evaluate the diagnostic ability of the ANN model in the training set and testing set.

### Construction of immune genes score

To quantify the expression patterns of immune-related genes in keloid patients, we constructed a scoring system to quantify the expression of immune-related genes and their diagnostic effects in keloid patients, which we called immune genes score (IG score).The process of establishing IG score is as follows:

First, we used the R package “ConensusClusterPlus” to perform unsupervised clustering of keloid patients; in terms of the clustering effect, the clustering stability was higher when k = 2. Thus, we characterized keloid patients into two different clusters (C1 and C2) based on the unsupervised clustering results. To construct the IG score, we performed principal component analysis (PCA) based on the KHIGs expression levels and used principal component 1 and principal component 2 as the feature scores.The formula for calculating the IG score is as follows:

### IG score=∑(PC1_i_ + PC2_i_)

where “i” stands for KHIG. we divided the samples with IG scores greater > 0 into the high IG score group, and the samples with IG scores < 0 into the low IG score group [29, 30].

### Evaluation of immune cell infiltration and their correlation with the KHIGs

Infiltration of immune cells was assessed using ssGSEA [31]. Specifically, ssGSEA was performed in R language using the R packages “GSVA” and “GSEABase” and the immunological characteristics of keloid patients were assessed using the ssGSEA algorithm, respectively. We performed single-sample gene set enrichment analysis (ssGSEA) and calculated ssGSEA scores. We used the “pheatmap” R package to visualize the infiltration levels of different immune cells. To assess the differential infiltration abundance of different immune cells between normals and keloids, we used the Wilcoxon test for pairwise comparisons.

### Fibroblast cells subtype recognition

We downloaded the scRNA-seq data of keloid from the previous study of *Deng et al.* [32], which contains 3 keloid samples, 3 control samples, and 40,655 cells, including 13,437 fibroblast cells after *Deng et al.* obtained the cells by quality control. We retrieved the scRNA-seq of 13,437 fibroblasts, which had been annotated, and analyzed them using the R package “Seurat“ [33]. Next, we normalized gene expression in fibroblasts using the “NormalizeData” function and performed principal component analysis using the “ElbowPlot” function to extract the top 20 principal components (PCs), which were further analyzed using the “FindVariableFeatures” function. We used the “FindNeighbors” and “FindClusters” functions for unsupervised and unbiased clustering of cell subpopulations. Then, we annotated fibroblast subpopulations using known markers [32], respectively PIF (APOE/CXCL3), MF (ASPN/POSTN), SRF (APCDD1/COL18A1), SPF (WISP2/MFAP5).

### Pseudotime trajectory analysis

Pseudotime trajectory analysis uses single-cell transcriptome sequencing to determine cell differentiation status. In the present study, we performed pseudotime trajectory analysis of individual cells using the R software package “monocle2“ [34–36]. We utilized the Seurat processed data as a matrix of Genes-Cells for raw UMI counts. We used the “newCellDataset” function to generate an object (expressionFamily = negbinomial. size) for pseudotime trajectory analysis. Subsequently, we included genes with an average expression value higher than 0.1 and detected their expression in at least ten cells for pseudotime trajectory analysis. We used the “reduceDimension” function to reduce the dimensionality of the cell differentiation trajectories with parameters reduction_method = “DDRTree” and max_components = 2. We used the “orderCells” function to construct pseudotime trajectories and order the cells, and we used “plot_cell_trajectory” to classify and visualize the cells.

### Identification and docking of drugs targeting KHIGs

Previous studies have shown that drug discovery begins with identifying disease targets, and target-based drug discovery is the most common strategy for new drug development [37, 38]. Thus, to identify drugs that can target KHIGs, we used the Enrichr platform (https://maayanlab.cloud/Enrichr/) for online identification and analysis. First, we entered the KHIGs gene symbols on the homepage of the Enrichr platform. Then, we screened the DSigDB database in the “Disease/Drugs” module to identify drugs that target KHIGs and set *p* < 0.05 as statistically significant. Subsequently, we investigated the interactions of the screened drugs with KHIGs using molecular docking techniques to identify the most promising drugs. Specifically, we obtained the molecular structures of the screened drugs from the PubChem database (https://pubchem.ncbi.nlm.nih.gov/). Meanwhile, the 3D coordinates of LIF (PDB ID, 1BQU; resolution, 2.00Å), PTGFR (PDB ID, 8IUK; resolution, 2.67 Å), and RBP5 (PDB ID, 7A9Y; resolution, 1.64 Å) were retrieved from the PDB database (https://www.rcsb.org/). Protein and molecular files were converted to PDBQT format, excluding water molecules, and including polar hydrogen atoms. To allow unrestricted molecular movement, a centered grid box surrounded the structural domains of each protein. Use the AutoDock tool for protein-ligand docking and visualize the interactions between receptors and ligands using PyMOL.

### Patients samples

In previous analyses, we found dysregulated expression of KHIGs in keloid scars. To further validate this finding, we collected samples from clinical patients in both disease and non-disease groups, and then validated KHIGs at the gene and protein levels (qPCR, WB and IHC). This study included three untreated keloid patients and three requiring skin grafting for abdominal skin removal. The detailed information is shown in Table S4. All patients signed the informed consent form, and samples (the full-thickness skin tissue) were collected, processed, and analyzed under the guidance of the Ethics Committee of Guangzhou Red Cross Hospital (Ethics Approval number 2022-292-01).

### Cell extraction and culture

Briefly, harvested tissue samples are washed with phosphate-buffered saline, cut into chunks, and digested with 1 mg/ml collagenase type I (SIGMA, USA). The digested tissue samples were centrifuged at 1500 × g for 5 min and filtered through a 100 μm filter. The supernatant was discarded, re-suspended in DMEM (CORNING, China), and cultured in DMEM supplemented with 10% fetal bovine serum (Gibco, Australia) at 37℃ and 5% CO_2_. The experiment used 2–3 primary cells.

### Eosin staining

Tissues were embedded in paraffin, sectioned into 4-µm intervals, and hydrated with a series of ethanol concentrations. The cleaned sections were placed in hematoxylin dye for about 5 min and eosin dye for 5 min. Then, the slices were dehydrated, sealed, observed, and photographed under microscopy (Nikon).

### Masson staining

Formalin-fixed paraffin-embedded skin tissue sections were stained with hematoxylin for 8 min, differentiated with 1% hydrochloric acid alcohol for 15 s, incubated with Masson blue solution for 15 s, and then subjected to Ponceau acid fuchsin staining for 5 min. After washing with 1% phosphomolybdic acid solution for 2 min, aniline blue staining was conducted for 2 min. The tissue sections were dehydrated with anhydrous ethanol three times for 10 s each, then incubated with xylene 3 times for 1 min each. Finally, the tissue sections were sealed with neutral resin, and microscopy (Nikon) was used to observe the stained skin tissues.

### Quantitative real-time polymerase chain reaction

Total RNA was extracted from keloid fibroblasts (KFb) and normal skin fibroblasts (HSFb) using Trizol (Ambion, USA). Then 500 ng RNA was used to synthesize cDNA with PrimeScriptTM RT Master Mix (Takara, Japan), and the mRNA levels were quantified using the TB GreenTM Premix Ex Taq II (Takara, Japan). The 2^−ΔΔCT^ relative quantitative algorithm analyzed the results. Primer sequence-Table S5.

### Western blot assay

Protein concentrations were determined by the BCA Protein Assay Kit (Thermo Fisher Scientific; USA) following HSFb and KFb cell lysis in RIPA buffer and PMSF (Beyotime; China). The 20ug protein sample was placed into a 10% or 8% sodium dodecyl sulfate-polyacrylamide gel for electrophoresis and then transferred to a polyvinylidene fluoride membrane (Merck; USA). The membrane was blocked for one hour at room temperature and incubated with primary antibodies overnight at 4℃. The primary antibodies used were Mouse anti-β-actin (1:1000, Servicebio, China). Rabbit anti-LIF (1:1000, Zenbio, China), Rabbit anti-RBP5 (1:1000, Zenbio, China), Rabbit anti-PTGFR (1:1000, Abcam, USA). The membranes were washed three times with 0.05% Tween 20 Tris-buffered saline and incubated with the corresponding HRP-conjugated secondary antibodies for two hours at room temperature. We measured the blots’ densities using an ECL reagent kit (Thermo Fisher, USA). The protein levels were detected using the ChemiDocTMXRS + imaging system (Bio-Rad; USA), which detected the protein expression. The data analysis uses ImageJ software. The internal control is β-actin.

### Immunohistochemistry assay

Formalin-fixed paraffin-embedded keloid and skin tissue are Dewaxed and rehydrated with dimethylbenzene and different concentrations of ethyl alcohol, then antigenically repaired and sealed. The sections were then incubated with the following primary antibodies overnight at 4℃: Rabbit anti-LIE (1:100, Zenbio, China), Rabbit anti-RBP5 (1:100, Zenbio, China), and Rabbit anti-PTGFR (1:400, Servicebio, China). Subsequently, the sections were rinsed thrice with phosphate-buffered saline for 5 min each time. The sections were then sequentially incubated with biotin-conjugated secondary antibodies and horseradish peroxidase (HRP)-conjugated secondary antibodies at 22℃ for 30 min. Chromogenic, hematoxylin staining. The samples were photographed by microscope (Nikon), and images were analyzed using ImageJ v2.1.0.

### Immunofluorescent assay

The sections of HSFb and KFb cells were treated with 0.3% Triton X-100 and blocked with 10% BSA. The processed sections were incubated overnight with primary antibodies at 4℃. The primary antibodies used were Rabbit anti-collagen I(1:200; Abcam, USA). The corresponding secondary antibodies (1:100; Zenbio, China) were added to the sections for specific binding. Phalloidin-SF488(Zenbio, China) was used to stain the actin filaments, and DAPI (Beyotime; China) was used to stain the nucleus. The stained sections were imaged using microscopy (Nikon).

### Statistical analysis

Statistical analysis and visualization were conducted using R and GraphPad Prism software for this study. The t-test and the Mann–Whitney U-test (the Wilcoxon rank sum test) were selected for whether the data conformed to a normal distribution. Statistical significance was defined as *p* < 0.05.

## Results

### Identification of differentially expressed immune-related genes in keloid

**Fig. 1:**
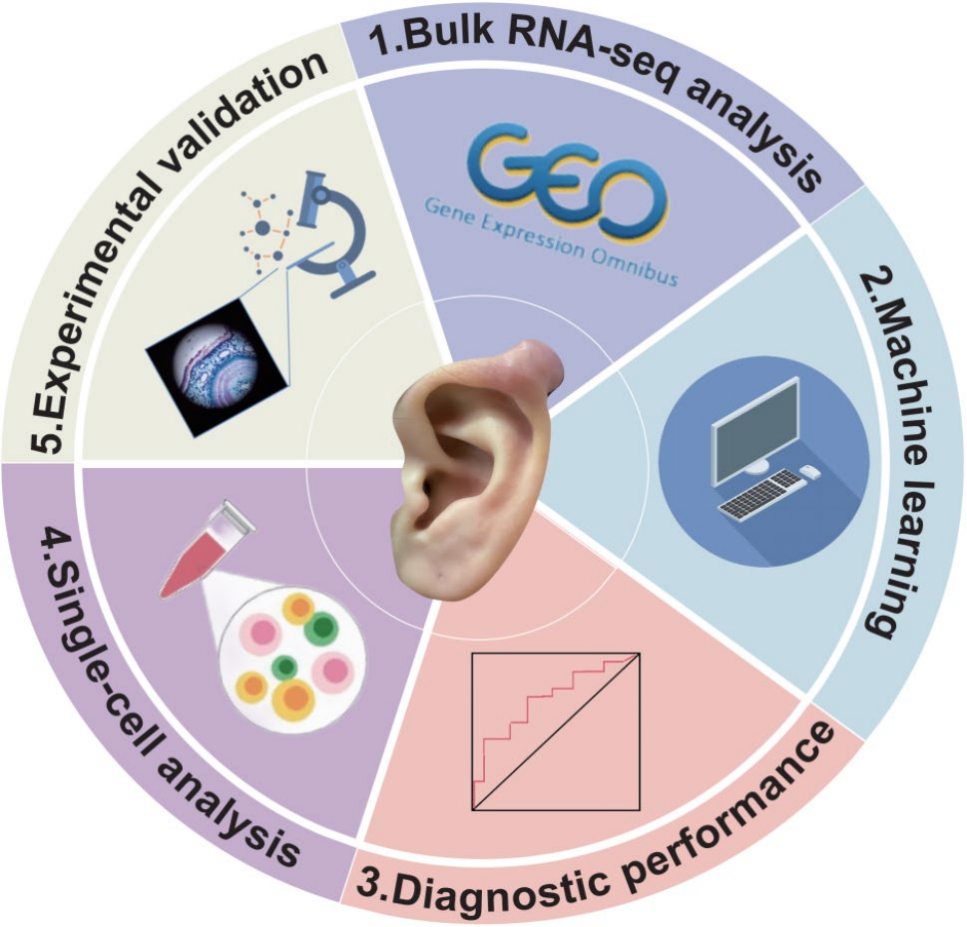
Schematic view of the procedures for data collection and analyses in keloid

**Fig. 2:**
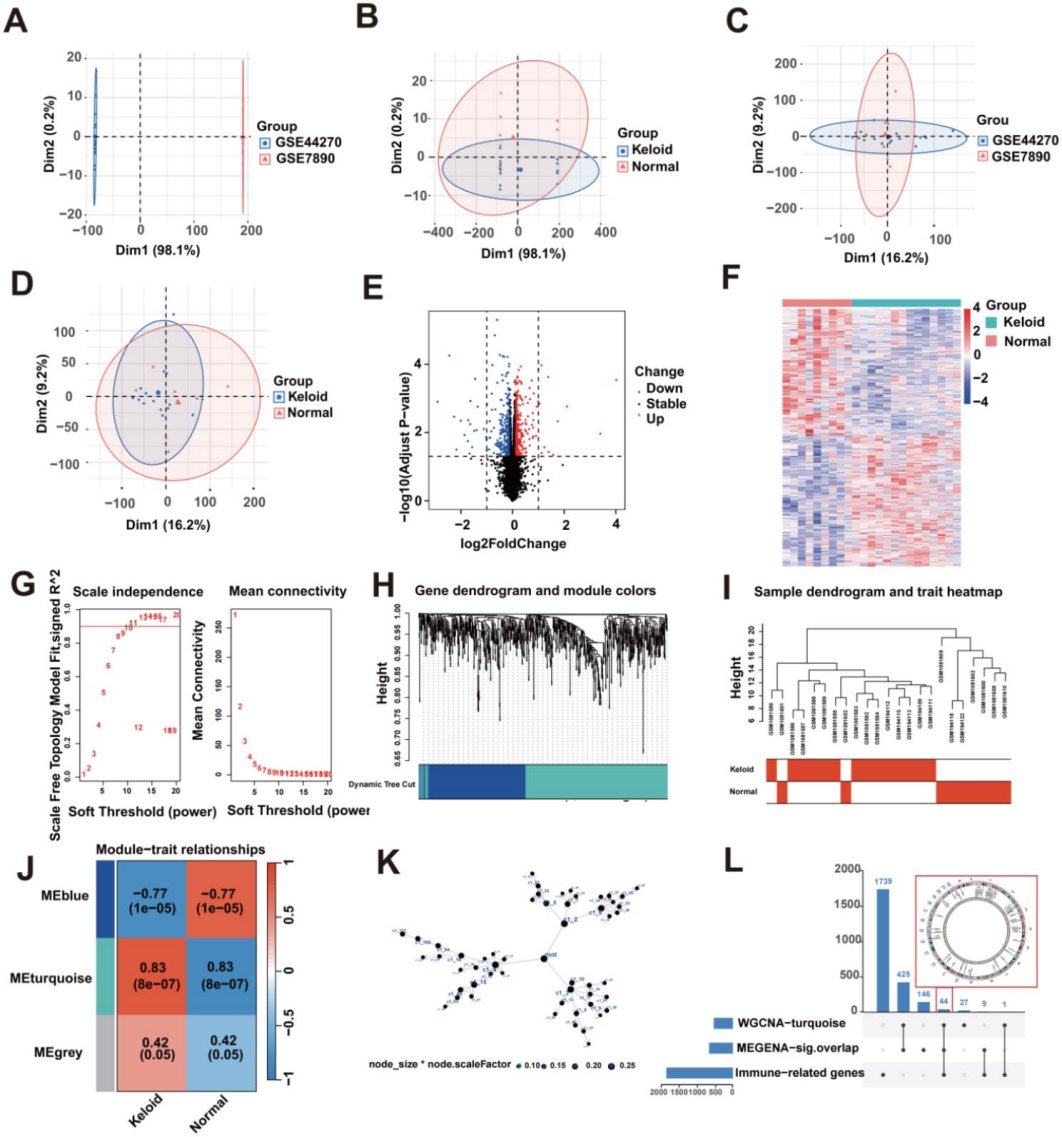
Identification of keloid/immune-related DEGs based on bulk RNA-seq. **(A-B)** The overall landscape of unprocessed data from the cohort. **(C-D)** The overall landscape of data from the cohort after removing batch effects. **(E)** Volcanic map showing the DEGs between keloids and controls. Red dots indicate up-regulated genes, and blue dots indicate down-regulated genes. **(F)** Heatmap showing the overall landscape of the DEGs between keloids and normals. **(G)** Graphs of the soft-threshold power versus scale-free topology model fit index and mean connectivity. **(H)** Dendrogram of the genes clustered based on a dissimilarity measure. **(I)** Dendrogram of samples and a heatmap plot of the indicated traits. **(J)** Analysis of the relationship between gene modules and traits (Keloid/Normal). **(K)** MEGENA analysis of DEGs, where each node represents a module and larger nodes indicate more genes. **(L)** The upset diagram showed crossover genes (keloid/immune-related DEGs) of WGCNA, MEGENA, and immune-related genes

We performed a series of analyses based on keloid bulk RNA-seq to systematically investigate the functions of immune-related genes in keloid, and the workflow chart of this study is shown in Fig. 1. However, different datasets exhibit data heterogeneities and batch effects. Figs. 2A and B illustrate the overall profiles of GSE44270 and GSE7890 before merging. We used the “ComBat” function in the R package “sva” to eliminate the batch effect [39]. Figs. 2C and D show the overall landscape of the GSE44270 and GSE7890 data after removing the batch effect using the ComBat function. The results indicated that the samples were uniformly distributed, the batch effect between GSE44270 and GSE7890 was removed, and the data could be used for subsequent analysis.

Following this, we obtained 836 DEGs by gene expression differential analysis (Table S6), of which 426 were significantly up-regulated and 410 were significantly down-regulated genes (Fig. 2E). The overall landscape of DEGs is shown in Fig. 2F. Subsequently, we subjected the 836 DEGs to WGCNA and MEGENA analyses to identify keloid-related DEGs. Specifically, we selected 10 as the soft threshold based on mean connectivity and scale independence (Fig. 2G). The hierarchical clustering of modules is carried out based on the topological overlap matrix (TOM), and similar modules on the cluster tree are merged (Fig. 2H). Cluster tree maps and characteristic heat maps of module feature genes were drawn (Fig. 2I). Lastly, we obtained three gene modules, and the results showed that the turquoise module was significantly positively correlated with keloids and had the highest correlation (*r* = 0.83, *p* = 8e-07) (Fig. 2J). Thus, we identified 497 genes relevant to keloids through the turquoise module (Table S7). Similarly, we also constructed the MEGENA network with 836 DEGs (Fig. 2K), and we identified a total of 53 modules by MEGENA analysis (Table S8), of which 25 modules had p value ≤ 0.05 (Fig. S1A), and the first four modules were shown as Fig. S1B. Eventually, the upset diagram showed 44 keloid/immune-related DEGs between WGCNA’s turquoise module, MEGENA’s significant module, and immune-related genes (Fig. 2L).

Meanwhile, we investigated the potential biological functions of the 44 overlapping DEGs based on GO and KEGG enrichment analyses, in which the GO results showed that they were mainly enriched in “chemokine receptor binding,” “growth factor receptor binding,” “vascular endothelial growth factor binding,” “extracellular matrix,” “transcription factor AP-1 complex”, “cell proliferation,” “apoptotic process” and “inflammatory response“ (Fig. S2A). The results of KEGG showed that they were mainly enriched in the” TNF signaling pathway,” “IL-17 signaling pathway”, “ErbB signaling pathway,” “TGF-beta signaling pathway,” “Chemokine signaling pathway,” “MAPK signaling pathway,” “Apoptosis” and “Jak-STAT signaling pathway“ (Fig. S2B).

#### Identification of KHIGs based on multiple machine learning algorithms

**Fig. 3:**
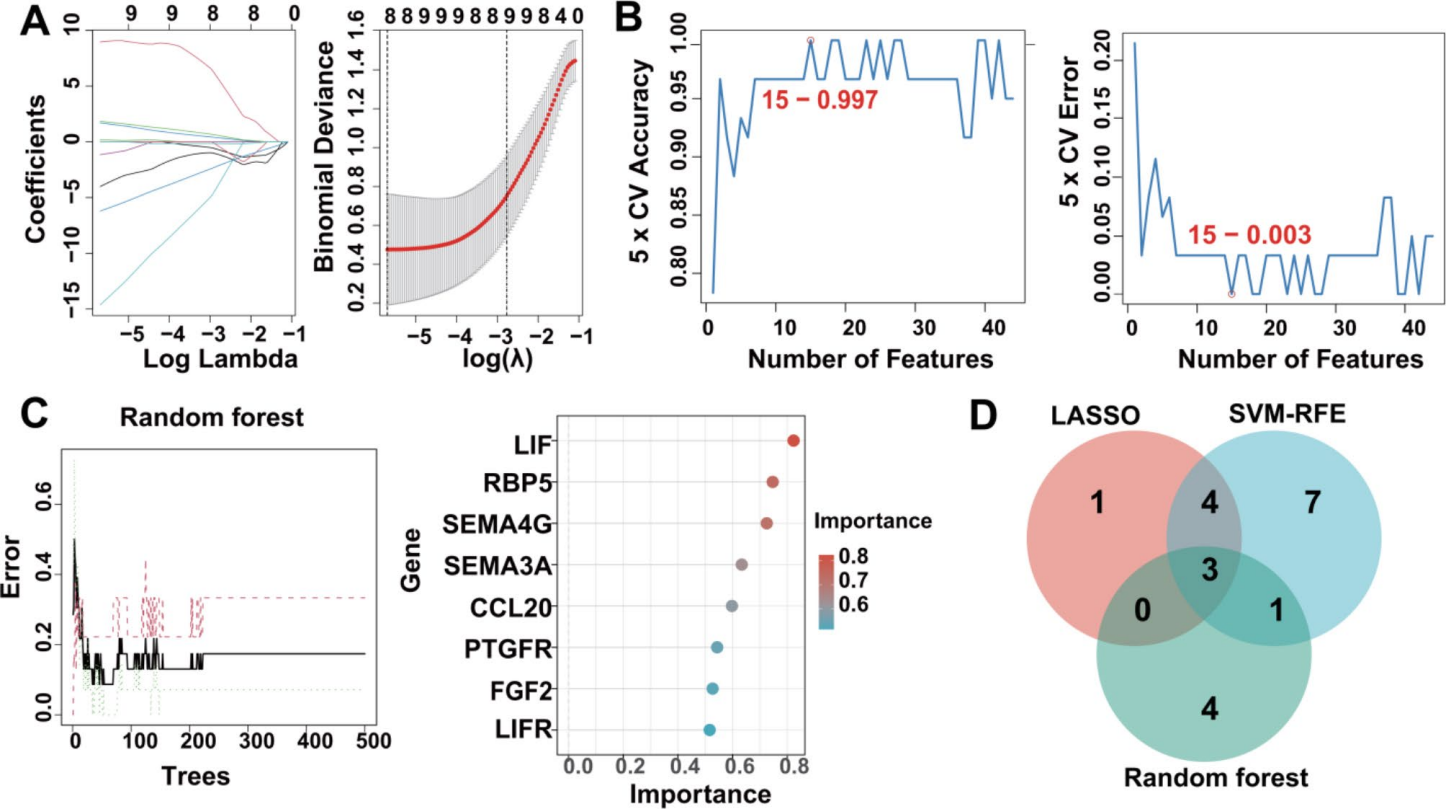
Identification of KHIGs using 3 machine learning algorithms. **(A)** LASSO coefficient profiles of the 44 overlapping DEGs (left panel). After cross-validation for tuning parameter selection, 8 candidate genes were identified (right panel). **(B)** SVM–RFE algorithm identified 15 candidate genes with an accuracy of 0.997 (left panel) and an error of 0.003 (right panel). **(C)** RandomForest algorithm identified 8 candidate genes. RandomForest error rate versus the number of classification trees (left panel) and gene importance scores (right panel). **(D)** Venn diagram showing the candidate genes (KHIGs) screened by RandomForest, SVM-RFE, and LASSO algorithms

To identify the hub immune genes in keloid, we applied three machine learning algorithms to analyze and identify the 44 keloid/immune-related DEGs, including LASSO, SVM-RFE and RF (Table S9). Specifically, we identified 8 candidate genes (ARG2, STC2, LIF, RBP5, C5, LEFTY2, GREM1, and PTGFR) by LASSO algorithm (Fig. 3A) and 15 candidate genes by SVM-RFE algorithm with an accuracy of 0.997 and an error rate of 0.003 (PTGFR, OPRD1, ARG2, GREM1, LIF, RBP5, C5, GAST, HSPA1L, ULBP1, GALR3, SEMA4G, LEFTY2, TNC, NEO1) (Fig. 3B), and RF algorithm identified 8 candidate genes with importance greater than 0.5 (LIF, RBP5, SEMA4G, SEMA3A, CCL20, PTGFR, FGF2, LIFR) (Fig. 3C). Subsequently, we intersected the candidate genes obtained from the mentioned above machine learning algorithms and finally found that PTGFR, RBP5, and LIF could be indicated by all the algorithms, meaning that PTGFR, RBP5, and LIF could be used as KHIGs for keloid under the multiplex algorithm (Fig. 3D).

#### Evaluation of the diagnostic performance of KHIGs applying to keloid

**Fig. 4:**
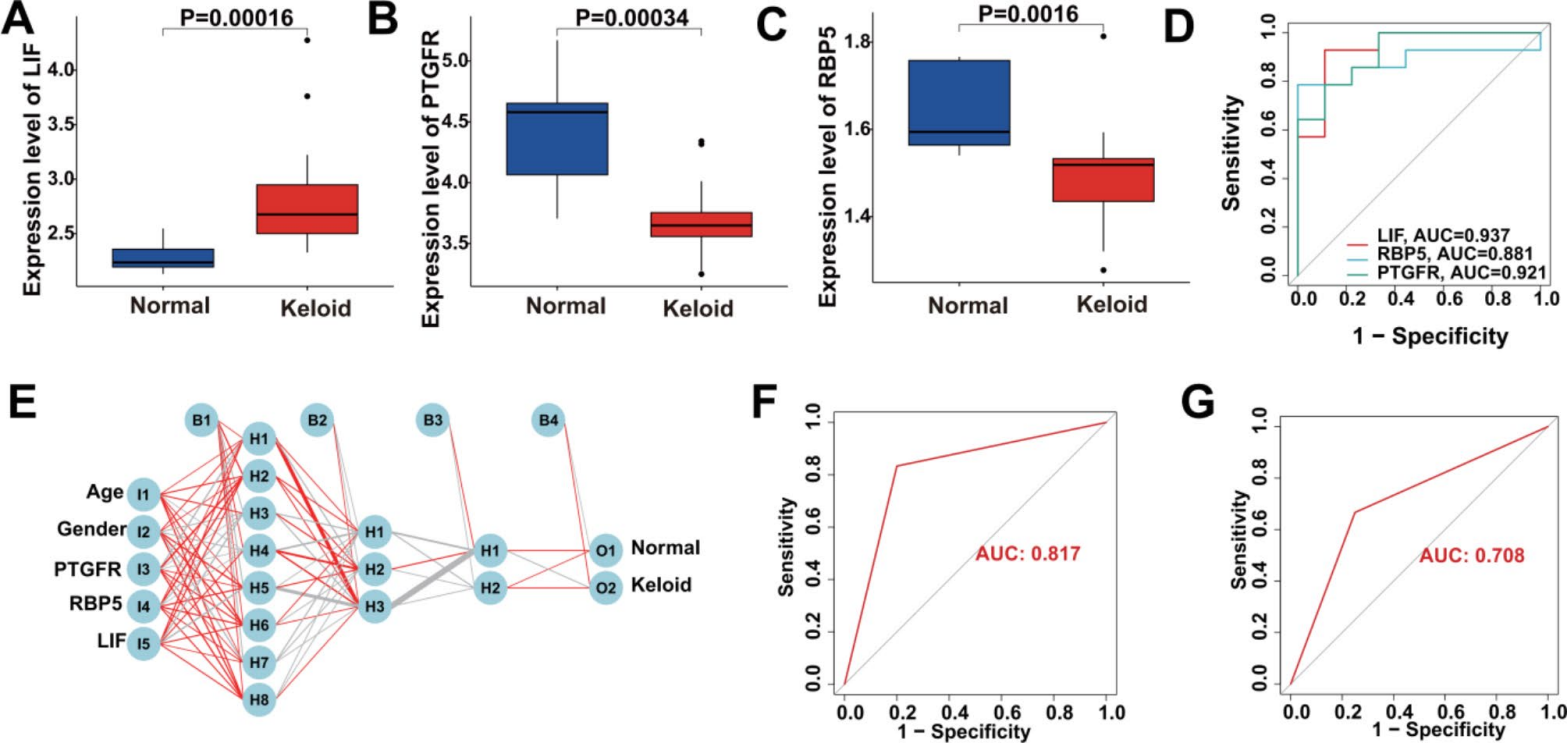
Diagnostic performance of KIHGs in keloids. (**A-C**) The expression of the screened KHIGs between the keloid and normal samples. **(D)** ROC showing the diagnostic performance of the screened KHIGs. **(E)** Construction of ANN based on the transcriptome level of KHIGs and the clinical characteristics of the samples (age and gender). **(F)** The AUC of the training set with a value of 0.817. **(G)** The AUC of the testing set with a value of 0.708

Immediately after that, we verified the importance of KHIGs in multiple dimensions. Specifically, based on the expression level, KHIGs were all dysregulated expression (Figs. 4A-C). Based on the diagnostic perspective, the receiver operating characteristic curve (ROC) showed that KHIGs all had a high area under the curve (AUC) values in which the AUC values of LIF, PTGFR, and RBP5 were respectively 0.937, 0.921, and 0.881 (Fig. 4D). Additionally, we also constructed an ANN model to diagnose the onset of keloids based on the transcriptome level of KHIGs and the clinical characteristics of the samples (age and gender) (Fig. 4E). Meanwhile, we assessed the prediction ability of the ANN using multiple evaluation metrics, including accuracy, precision, recall, F1-score and AUC. The results of the ANN predicting training and testing sets are shown in Table 2; Figs. 4F and G, where the accuracy, precision, recall, F1-score and AUC of the training set are 0.813, 0.890, 0.800, 0.842 and 0.817, respectively, and those of the testing set are 0.714, 0.750, 0.750, 0.750 and 0.708, respectively. Overall, the ANN model was convincing as an independent diagnostic predictor of keloids.

**Table 2:** ANN diagnosis effect for the training and testing set

		Training set		Testing set	
		Normal	Keloid	Normal	Keloid
Prediction	Normal	5	2	2	1
	Keloid	1	8	1	3
	Accuracy	0.813		0.714	
	Precision	0.890		0.750	
	Recall	0.800		0.750	
	F1-score	0.842		0.750	
	AUC	0.817		0.708	

#### Construction and validation of the IG score based on KHIGs

**Fig. 5:**
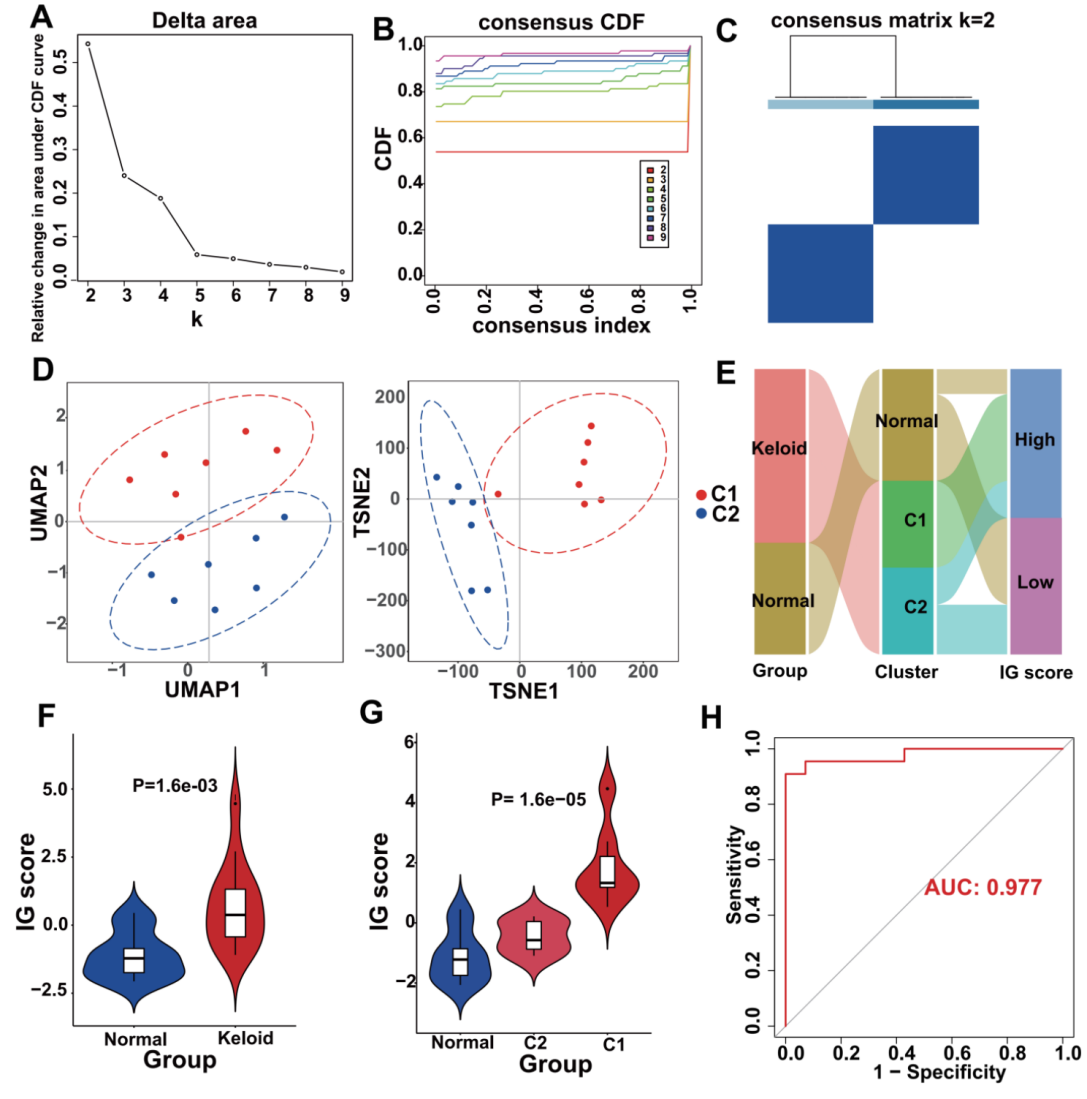
Construction of IG scores for assessment of keloid risk. (**A-C)** Unsupervised clustering of keloids based on KHIGs. **(D)** UMAP (left panel) and t-SNE (right panel) analyses to validate the effect of unsupervised clustering of keloids by KHIGs, with each point representing a sample. **(E)** Alluvial plots showing the associations between the three groups: disease state grouping, unsupervised clustering grouping and IG score grouping. **(F-G)** Violin plots showing the comparison of IG scores between the normals and the keloids distributed in C1 and C2. **(H)** The ROC curve used to evaluate the IG score

To assess the correlation between KHIGs and keloid risk, we performed unsupervised clustering of keloid patients (Figs. 5A-C). The heatmap showed clear boundaries and stable and reliable clustering of the cluster1 and cluster2 (C1 and C2) when k = 2, and the Uniform Manifold Approximation and Projection (UMAP) and t-distributed stochastic neighbor embedding (t-SNE) dimensionality reduction analyses performed also showed that C1 and C2 were well-dispersed (Fig. 5D). Subsequently, we calculated the IG score based on the KHIGs and further assessed the risk of keloid in each sample using principal component analysis, and all samples were divided into low or high IG score subgroups based on IG scores < or > 0. The results showed that the high IG score subgroup belonged to C1, whereas the low IG score subgroup belonged to C2 and the normal group (Fig. 5E). We also compared the IG score of the normal, C1 and C2 separately, and the results showed that the C1 had higher IG scores than the C2 and the IG scores of keloid (C1 and C2) were higher than the normal group (Figs. 5F and G). Lastly, we performed the ROC analysis to further assess the predictive ability of the IG score, and the results showed that the AUC value of IG score was 0.977, which means that it has a good predictable performance (Fig. 5H). In conclusion, these data not only suggest that IG score based on KHIGs are predictive in identifying the risk of keloid patients, but also imply that keloids themselves may be heterogeneous and have different disease subtypes.

### Immune cell infiltration analysis

**Fig. 6:**
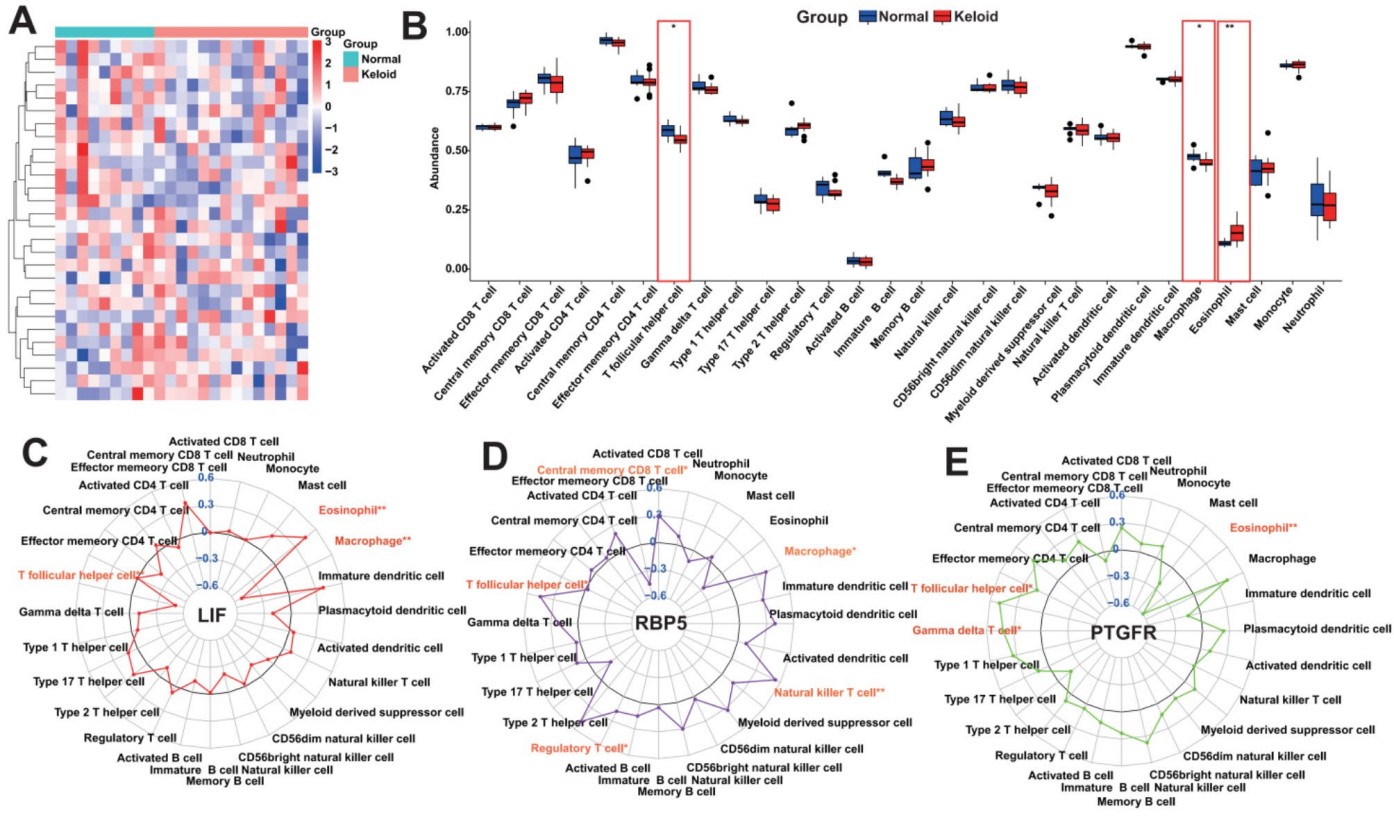
Association of immune cell infiltration abundance with KHIGs. **(A)** Heatmap showing the overall landscape of immune cell abundance for keloid samples and normal samples. **(B)** Box plots showing the differences in immune cell infiltration abundance between keloid samples and control samples. **(C)** Correlation analysis between the expression of LIF and immune cell infiltration abundance. On the outside of the thick black line it is a positive correlation and on the inside of the thick black line it is a negative correlation. **(D)** Correlation analysis between the expression of RBP5 and immune cell infiltration abundance. On the outside of the thick black line it is a positive correlation and on the inside of the thick black line it is a negative correlation. **(E)** Correlation analysis between the expression of PTGFR and immune cell infiltration abundance. On the outside of the thick black line it is a positive correlation and on the inside of the thick black line it is a negative correlation. (ns, no significance, *p < 0.05, **p < 0.01, ***p < 0.001)

We performed evaluation of immunological characterization of keloid samples using the ssGSEA algorithm, that is, we calculated the of immune cells abundance for each sample, including normal samples. Fig. 6A showed the overall immune cell infiltration, and the results indicated that there was a significant difference in immune cell infiltration between the keloids and the normals. Compared with normals, keloids have lower “T follicular helper cell” and “Macrophage” (Fig. 6B). Following this, we investigated the relationship between the KHIGs expression and the abundance of the immune cells mentioned above that are dysregulated in abundance by correlation analysis. We found significant correlations between the KHIGs expression and the abundance of these immune celltypes. For example, the LIF expression was negatively correlated with the abundance of “T follicular helper cell” and “Macrophage” (Fig. 6C), whereas the RBP5 expression was negatively correlated with its abundance (Fig. 6D), and then the PTGFR expression was positively correlated with the abundance of “T follicular helper cell” and “Gamma delta T cell” (Fig. 6E). The significant correlation between the KHIGs expression and the immune cell abundance implies that KHIGs may have a potential role in regulating the immune microenvironment of keloid.

### Single-cell RNA seq analysis of skin tissue

**Fig. 7:**
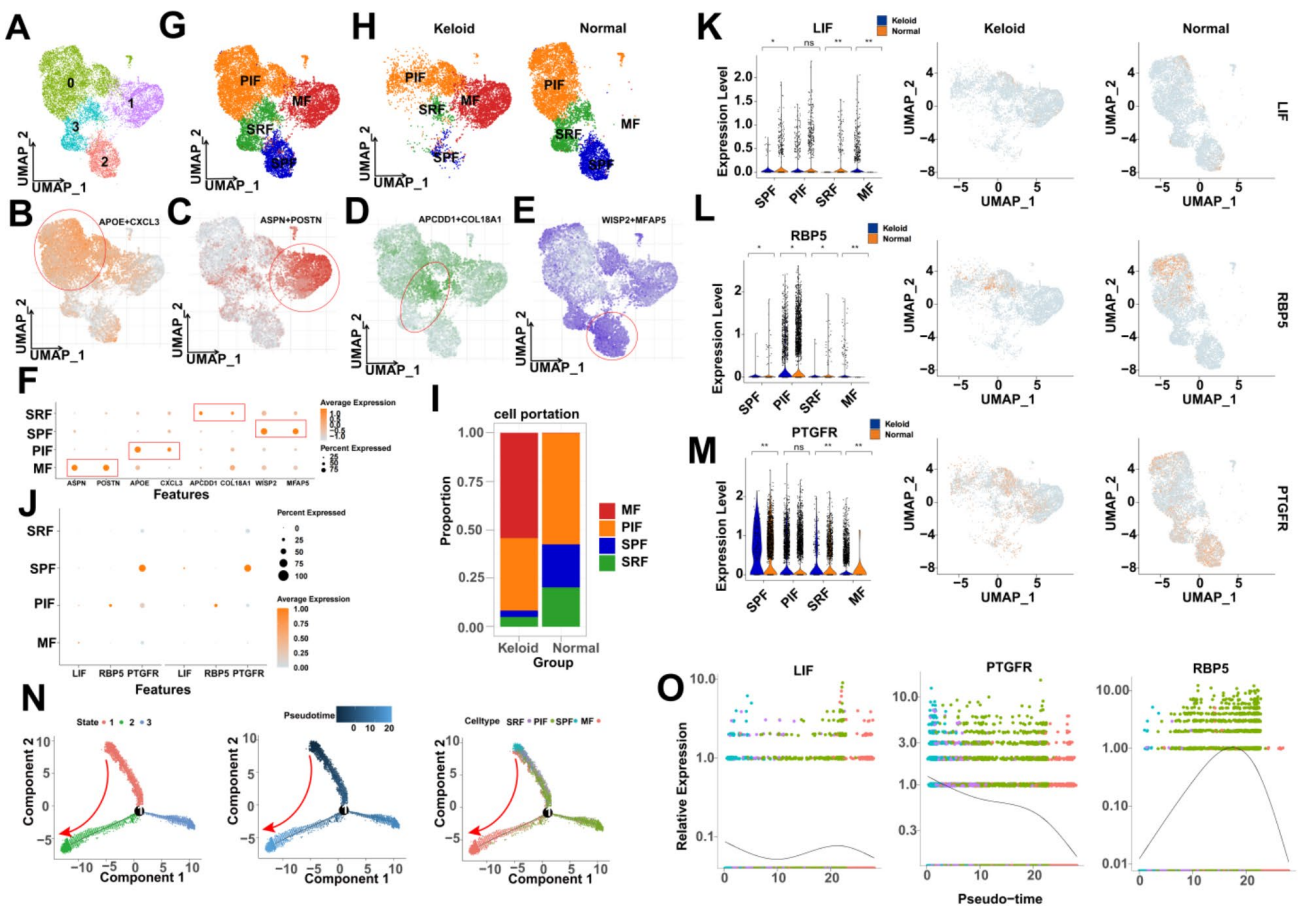
Single-cell RNA seq analysis of skin tissue. **(A)** 4 cell clusters were identified. **(B-E)** Heatmap of known marker expression in each type of cell subpopulation. **(F)** Dot plot of known marker expression in each type of cell subpopulation. **(G-H)** Mesenchymal fibroblast (MF), pro-inflammatory fibroblast (PIF), secretory-papillary fibroblast (SPF), and secretory-reticular fibroblast (SRF), the 4 types of fibroblast subtypes were annotated by known markers. **(I)** Counting of cell proportions. **(J)** Dot plot of KHIGs expression in the four types of fibroblast subtypes. **(K-M)** Violin plots (left panels) and heatmaps (right panels) of KHIGs expression in four types of fibroblast subtypes; each dot represents one cell. **(N)** Differentiation trajectories of fibroblasts, respectively, grouped based on differentiation status, pseudotime sequence, and cell types. **(O)** Jitter plot of KHIGs heterogeneously expressed with cell differentiation (pseudotime sequence). (ns, no significance, *p < 0.05, **p < 0.01, ***p < 0.001)

Single-cell seq of 13,437 fibroblasts from 3 keloid samples and 3 normal samples were integrated into the present study (Table S10), and four clusters were identified based on principal component analysis and the UMAP algorithm (Fig. 7A). We annotated the fibroblast subtypes according to the known markers [32], that is, PIF (APOE/CXCL3), MF (ASPN/POSTN), SRF (APCDD1/COL18A1), SPF (WISP2/MFAP5) (Figs. 7B-F). The single-cell landscapes of annotated fibroblast subtypes are shown in Figs. 7G and H. We found that MF and PIF accounted for a higher proportion of the various fibroblast subtypes by cell percentage counting, with MF being more prevalent in keloids and PIF being more prevalent in normal tissues (Fig. 7I). Lastly, we have shown the KHIGs expression in fibroblast subtypes in Figs. 7J-M.

Fibroblasts exhibit temporal heterogeneity in gene expression patterns during differentiation. To explore the heterogeneous expression of KHIGs during fibroblast differentiation, we used the “Monocle2” R package to perform pseudotime analysis. The pseudotime analysis of fibroblasts showed one node and three states during fibroblast differentiation, which were attributed to pseudotime values for the differentiation process. The results according to differentiation state, cell type, and pseudotime sequence showed that fibroblasts differentiated chronologically from the beginning to the end of the differentiation trajectory in state 1, state 3, and state 2, which means that PIF, SPF, and SRF tend to differentiate towards MF (Fig. 7N). Subsequently, we found that the expression pattern of KHIGs on fibroblast differentiation trajectories also showed temporal heterogeneity; that is, LIF showed a tendency to decrease, then increase, and then decrease, and the expression of PTGFR gradually decreased, whereas the expression of RBP5 showed a tendency to increase and then decrease (Fig. 7O).

## Validation of KHIGs

**Fig. 8:**
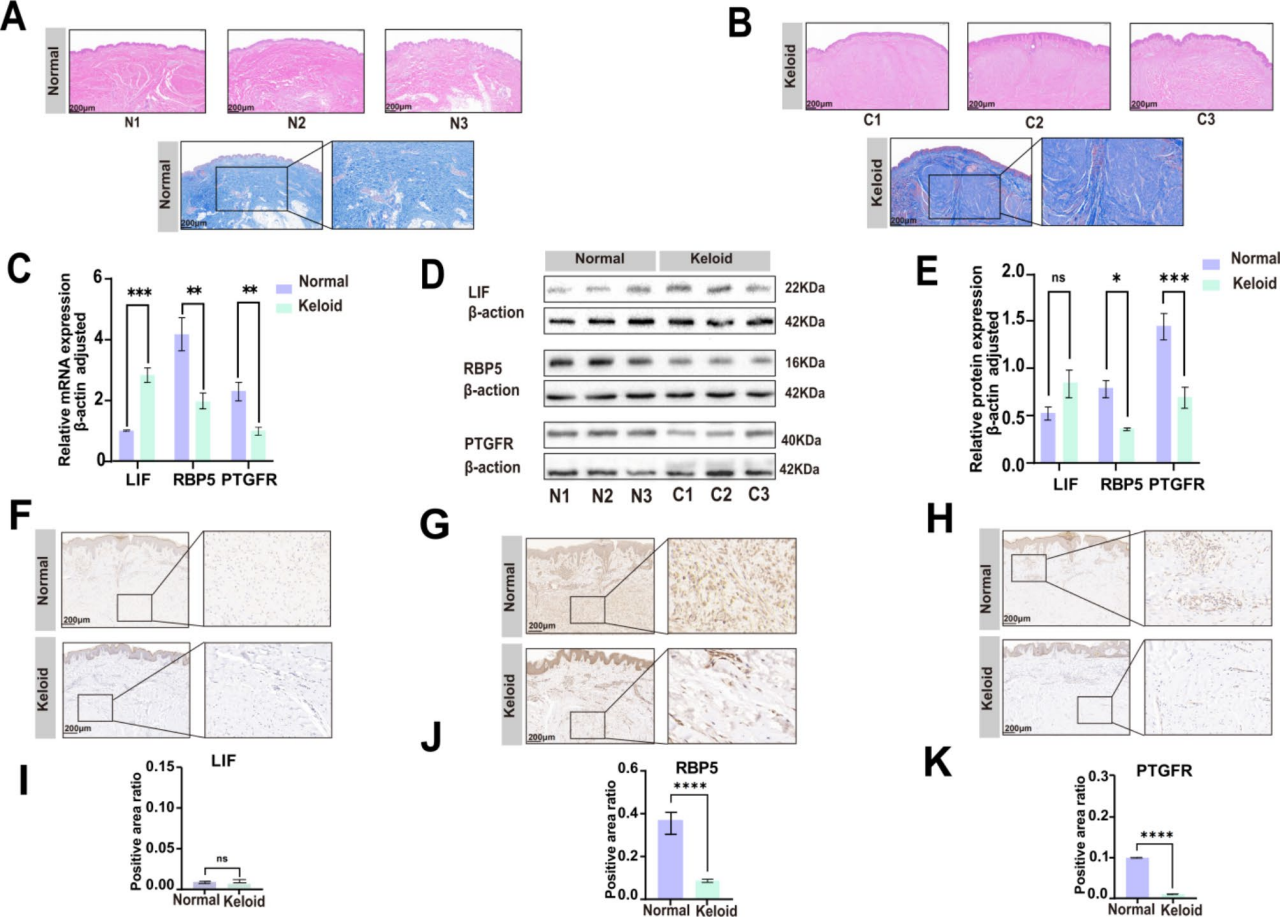
Validation of KHIGs. **(A-B)** HE and Masson staining of normal skin and keloid. **(C)** Relative mRNA expression levels of KHIGs. **(D)** Western blotting assay of KHIGs, it is related to Figure S3. **(E)** Relative protein levels of KHIGs. **(F-H)** Immunofluorescent assay of KHIGs. **(I-K)** Relative protein levels of KHIGs. (ns, no significance, *p < 0.05, **p < 0.01, ***p < 0.001)

The HE and Masson stain can show the difference between keloids and normal skin lesions. In normal skin, the fibers are arranged regularly and densely. In keloid, more fibers are found in the dermis, arranged irregularly and densely (Figs. 8A and B). To verify the expression of KHIGs in keloid, mRNA and protein expression levels of fibroblasts in keloid and normal skin were detected by qRT-PCR and Western blot, and KHIGs protein levels in keloid and normal skin were detected by IHC. The results of qRT-PCR showed that KHIGs had significant differences in keloid fibroblast samples (p value ≤ 0.05) (Fig. 8C), and LIF expression was up-regulated in keloid. At the same time, PTGFR and RBP5 were down-regulated in keloid. Western blot (Figs. 8D and E) and IHC (Figs. 8F-K) showed that PTGFR and RBP5 verified our results at the protein level (p value ≤ 0.05), and LIE expression was less and did not differ at the protein level.

### Evaluation of retinoic acid

**Fig. 9:**
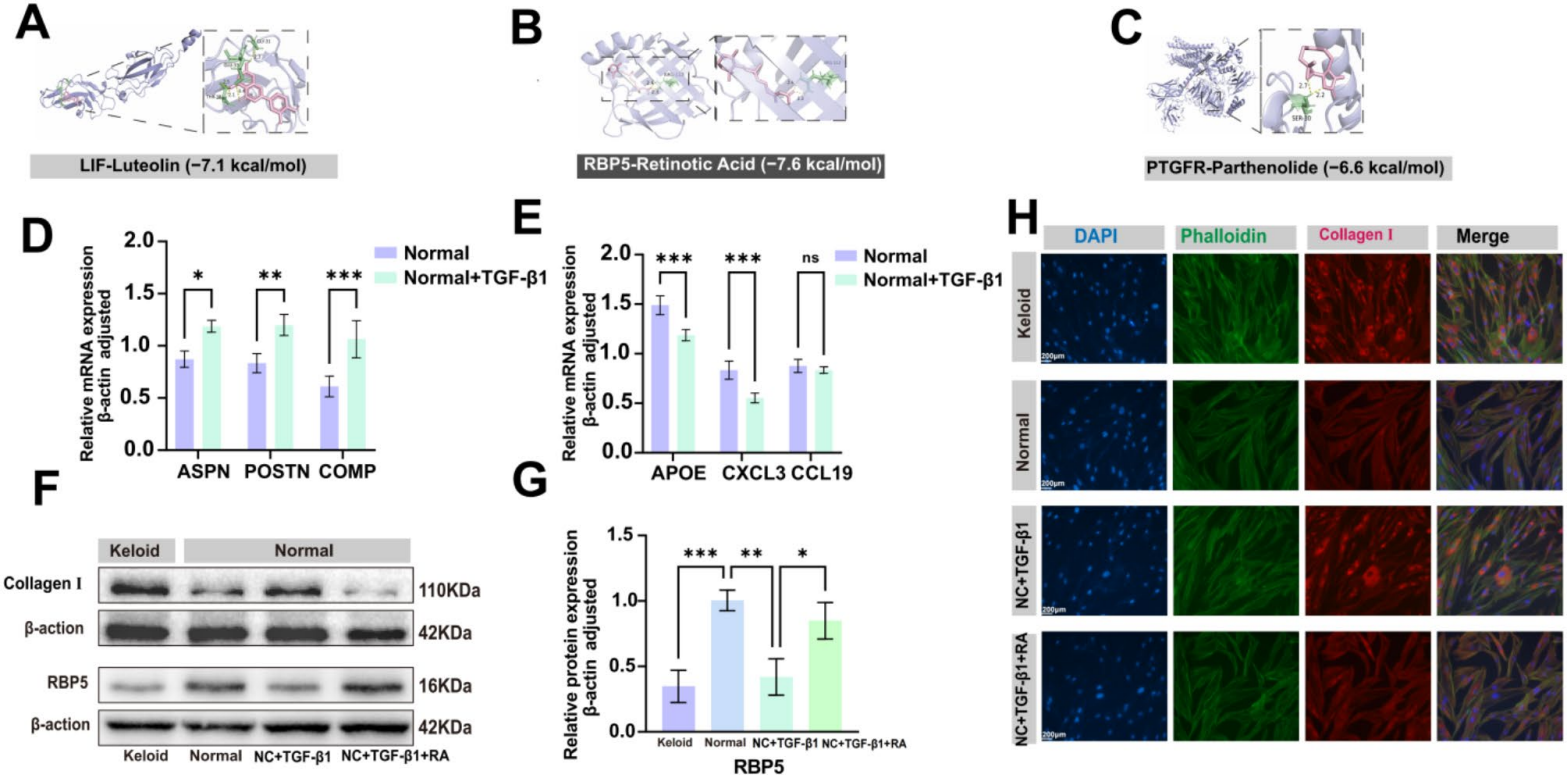
Retinoic Acid inhibition of TGF-β1-induced collagen I production in fibroblasts. **(A)** The structure of the complex is formed by the docking of Luteolin with LIF. **(B)** The structure of the complex is formed by the docking of Retinoic acid with RBP5. **(C)** The structure of the complex is formed by the docking of Parthenolide with PTGFR. **(D)** Relative mRNA expression levels of ASPN, POSTN, and COMP. **(E)** mRNA expression levels of APOE, CXCL3, and CCL19. **(F)** Western blotting assay of RBP5 and collagen I, it is related to Figure S3. **(G)** Relative protein levels of RBP5 and collagen I. **(H)** Immunofluorescent assay of Collagen I, DAPI, and Phalloidin. (ns, no significance, *p < 0.05, **p < 0.01, ***p < 0.001)

We used the Enrichr platform to identify potential drugs by targeting KHIGs that can be used to treat or alleviate keloids. We found that Luteolin(LIF) (Fig. 9A), Retinoic acid (RBP5) (Fig. 9B), and Parthenolide (PTGFR) (Fig. 9C) bind closely (p value ≤ 0.05). To analyze the binding affinity of the three drugs, the MMPBSA method was used to calculate the binding free energy of drugs and molecules. The results showed that KHIGs were all able to dock with the identified drugs with high binding energies, such as LIF-Luteolin (-7.1 kcal/mol), RBP5-Retinoic acid (-7.6 kcal/mol), PTGFR- Parthenolide (-6.6 kcal/mol). In summary, it is suggested that these drugs may positively affect the targeted treatment of keloid. To explore the role of Retinoic acid in the pathogenesis of keloid. First, we will treat HSF cells with 10µM TGF-β1 for 48 h. We found that the mRNA expression of MF (ASPN, POSTN, and COMP) was up-regulated (p value ≤ 0.05), and the mRNA expression of PIF (APOE, CCL19, and CXCL3) was down-regulated(p value ≤ 0.05) (Figs. 9D and E). They indicate that TGF-β1 could further increase the proportion of MF in normal skin fibroblasts. Then, 1µM Retinoic acid was added to 10µM TGF-β1 and treated together for 48 h. The results showed (Figs. 9F-H) that 10µM TGF-β1 group protein RBP5 expression decreased, and collagen type I significantly increased compared with the control group. More interestingly, after adding Retinoic acid to TGF-β1, we found that Retinoic acid can promote the increase of RBP5 expression and inhibit the production of collagen I.

**Fig. 10:**
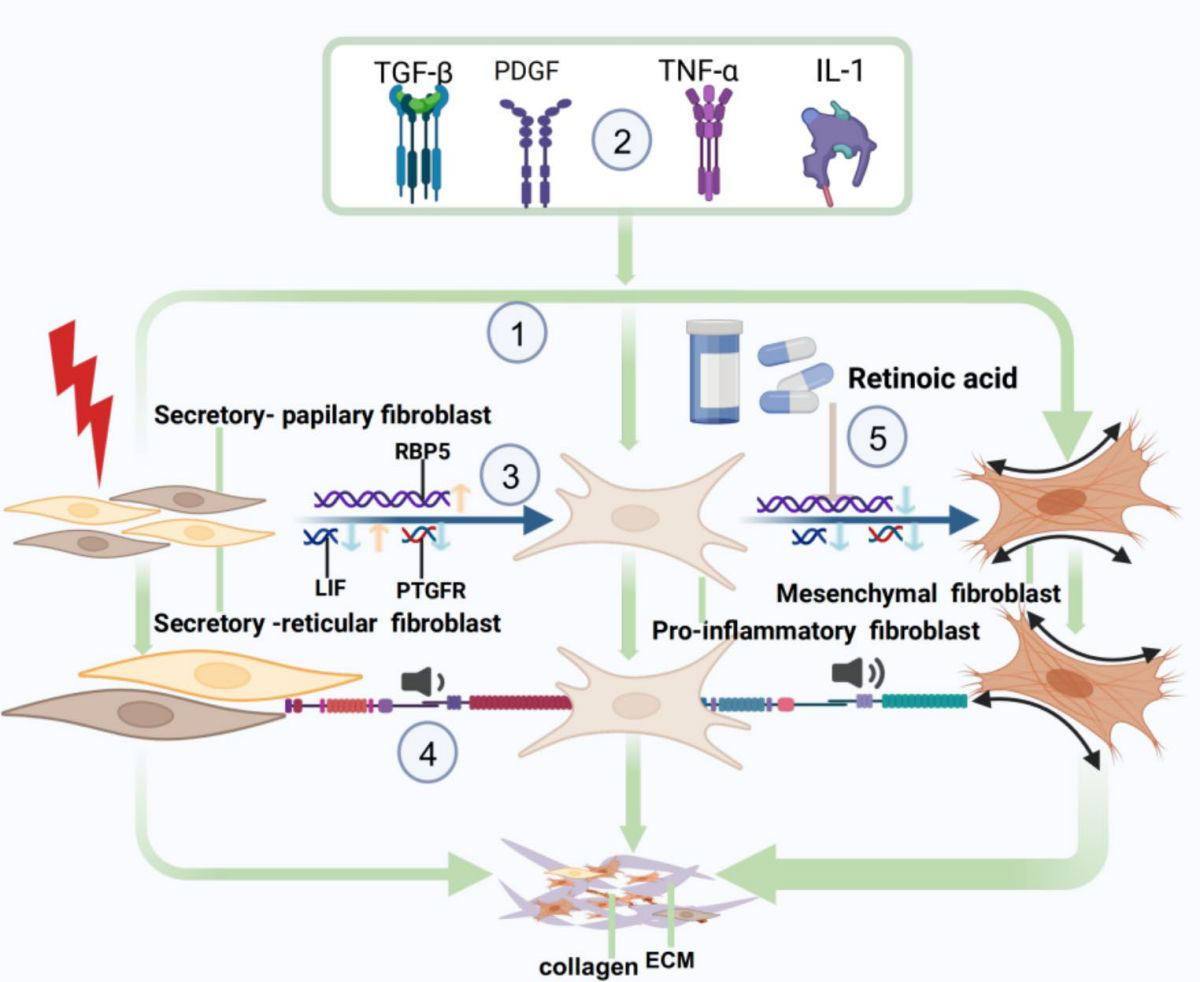
Schematic illustration illustrating the pathogenesis of keloid

### The potential regulatory mechanism of KHIGS in contributing to keloid

To reveal the mechanism of KHIGS in keloid, we further integrated the literature reports [32, 40] and our research results. We drew the mechanism diagram of KHIG in keloid: (1) Fibroblast differentiation trajectory: the trend of PIF, SPF, and SRF differentiation to MF. (2) TGF-β, TNF-α, PDGF, and Wnt signaling pathways play a key role in regulating fibroblast function. (3) KHIGs also showed temporal heterogeneity in the expression pattern of fibroblast differentiation locus; that is, LIF decreased first and then increased, and PTGFR expression decreased first and then gradually decreased. The expression of RBP5 decreased first and then increased. (4) Communication between SPF and SRF cells and PIF decreased, while communication between PIF and MF cells increased. (5) Retinoic acid further inhibits the transformation of PIF to MF by promoting the expression of RB5, thereby reducing the production of collagen and extracellular matrix (ECM) (Fig. 10).

## Discussion

Keloids result from abnormal scarring on injured skin and are characterized by excessive proliferation of fibroblasts, excessive production of extracellular matrix, and excessive deposition of collagen, often causing physical and psychological distress to the patient [41, 42]. At present, treatments for keloids, such as surgery, radiation, and physical therapy, are ineffective and have a high recurrence rate [43]. In keloid pathogenesis, the accumulation of many inflammatory cells in keloid lesions, such as macrophages, mast cells, and T lymphocytes, suggests that keloid lesions may be an inflammatory skin disease [44]. Hence, most current research has focused on the inflammatory aspects of keloids [45]. Here is little emphasis on the importance of immunity for keloids. There is a new view that immunity plays a crucial role in preventing pathogen invasion, inducing inflammation, and regulating fibroblasts in keloid in the study of keloid mechanism [46]. Thus, it is necessary to study keloids in immune-related aspects through bioinformatics.

In the present study, we first obtained 836 DEGs, including 410 up-regulated genes and 426 down-regulated genes, from publicly available datasets of keloid and normal skin tissue samples after removal of batch effects. Then, we intersected the keloid-related genes identified by WGCNA and MEGENA analyses with the immune gene list and finally obtained 44 keloid-related immune genes. We performed GO and KEGG enrichment analyses on the mentioned 44 genes, and GO analyses showed that these genes were mainly involved in inflammatory responses, cell proliferation, apoptosis, and other processes in biological processes, which was consistent with the results of previous studies [47–49]. Regarding cellular components, these genes were mainly enriched in the extracellular matrix, transcription factor AP-1 complex, collagen-containing extracellular matrix, etc., consistent with previous studies [50–52]. Regarding molecular functions, these genes were mainly enriched in chemokine receptor binding, growth factor receptor binding, integrin binding, etc., which was consistent with previous studies [53–55]. KEGG analysis showed that 44 keloid-related immune genes were mainly enriched in the Jak-STAT signaling pathway, MAPK signaling pathway, TGF-β signaling pathway, and other related signaling pathways, consistent with previous studies [56–58]. These results suggest that 44 keloid-related immune genes may regulate these key signaling pathways during the development and progression of keloids, which needs to be further investigated.

We further screened 44 keloid-related immune genes using multiple machine-learning algorithms: LASSO, SVM-RFE, and RF. We showed that LIF, PTGFR, and RBP5 can be indicated by all algorithms named KHIGs. LIF was significantly up-regulated in keloids compared to normal skin, while PTGFR and RBP5 were down-regulated in keloids. In addition, our ANN models constructed based on the expression of KHIGs and the clinical information of the samples all had AUC values higher than 0.7. Previous studies have shown that the area under the ROC curve is greater than 0.5, proving that the diagnostic model has some diagnostic value [59]. That means that KHIGs have good performance as dysregulated immune genes associated with keloid in predicting keloid. Notably, the leukemic inhibitory factor (LIF) is the most variable member of the interleukin-6 family [60], and LIF has previously been shown to be a potential therapeutic target for renal interstitial fibrosis [61]. The prostaglandin F(2α) receptor (PTGFR) is thought to play a role in pregnancy and labor [62], while Retinol-binding protein 5 (RBP5) regulates cell differentiation and proliferation [63].

It is well-known that fibroblasts are crucial factors in the pathogenesis of keloids. Previous studies have shown four different subtypes of fibroblasts in keloids and normal tissues [64]. We further found that the trend of KHIGS expression was also different in different cell subpopulations, which may suggest that the heterogeneous expression of KHIGS in different cell subtypes may also promote keloid. In addition, in our pseudo-time analysis, MF belonged to terminally differentiated cells. In contrast, fibroblasts of the other three subtypes could be transformed into MF. This further suggests that KHIGS plays a sophisticated function in the molecular mechanisms of keloids, implying that the heterogeneous expression of KHIGS in different fibroblast subtypes epitomizes the heterogeneity of keloids, meaning that KHIGS may serve as a predictor of keloid risk.

It has been shown in previous studies that the process of drug discovery begins with the identification of disease targets, and target-based drug discovery is the most common strategy for new drug exploitation [37, 38]. Current data mining of existing biomedical data and information contributes significantly to target discovery in the “Omics” era. This implies that target discovery is a critical step in the biomarker and drug discovery process for diagnosing and treating human diseases [65–67]. In the present study, we identified three potential drugs by targeting KHIGS: Parthenolide (PTGFR), Luteolin (LIF), and Retinoic acid (RBP5). Parthenolide, naturally occurring sesquiterpene lactone derived from feverfew, exhibits exceptional anti-cancer and anti-inflammatory properties [68]. The research suggests that Parthenolide could inhibit the UVB-induced proliferation of keratinocytes and melanocytes in the mouse skin [69]. Luteolin is a flavonoid found in different plants, such as vegetables, medicinal herbs, and fruits, as a modulator of skin aging and inflammation [70]. As a modulator of skin aging and inflammation, it has been extensively studied in skin aging, skin cancer, wound healing, and inflammatory skin diseases [71]. Retinoic acid is a metabolic product derived from vitamin A, acting at a nuclear level to maintain the proper transcriptional activity [72]. Retinoic acid and its derivatives have therapeutic potential for severe skin diseases [73]. Based on the above studies, it can be seen that the above three drugs have positive effects on skin diseases. We then used molecular docking methods to investigate further the binding affinity between these drugs and their related KHIGS, so we speculate that Parthenolide, Luteolin, and Retinoic acid are potential drugs for treating keloids. Finally, we chose retinoic acid with the highest binding energy. Previous studies have shown that retinoic acid can treat keloids [74], but the specific mechanism is unknown. In the experiment, we believe that retinoic acid may inhibit the form of PIF to MF differentiation through RBP5 to reduce collagen secretion further.

## Conclusion

In conclusion, the present study provided new immune signatures (PTGFR, RBP5, and LIF) for keloid diagnosis and treatment using multiple bioinformatic analyses and machine learning algorithms. Meanwhile, retinoic acid was identified as a potential anti-keloid drug by molecular docking methods as well as experimental validation. It helps to break through the current dilemma encountered in the diagnosis and treatment of keloid in the clinic.

### Electronic supplementary material

Below is the link to the electronic supplementary material.

Supplementary Material 1

Supplementary Material 2

## Data Availability

Datasets related to this article are from public database (GSE44270, GSE7890 and GSE163973). All data generated or analyzed during this study are included in this article/Additional files, further inquiries can be directed to the corresponding author.
