# Supplementary material for: Identification of novel immune-related signatures for keloid diagnosis and treatment: insights from integrated bulk RNA-seq and scRNA-seq analysis
Source: Hum Genomics. 2024 Jul 16;18:80. doi: 10.1186/s40246-024-00647-z (PMC11251391; doi:10.1186/s40246-024-00647-z)
Supplement: Supplementary file 2 — Supplementary Material 2 [file 40246_2024_647_MOESM2_ESM.docx]

**
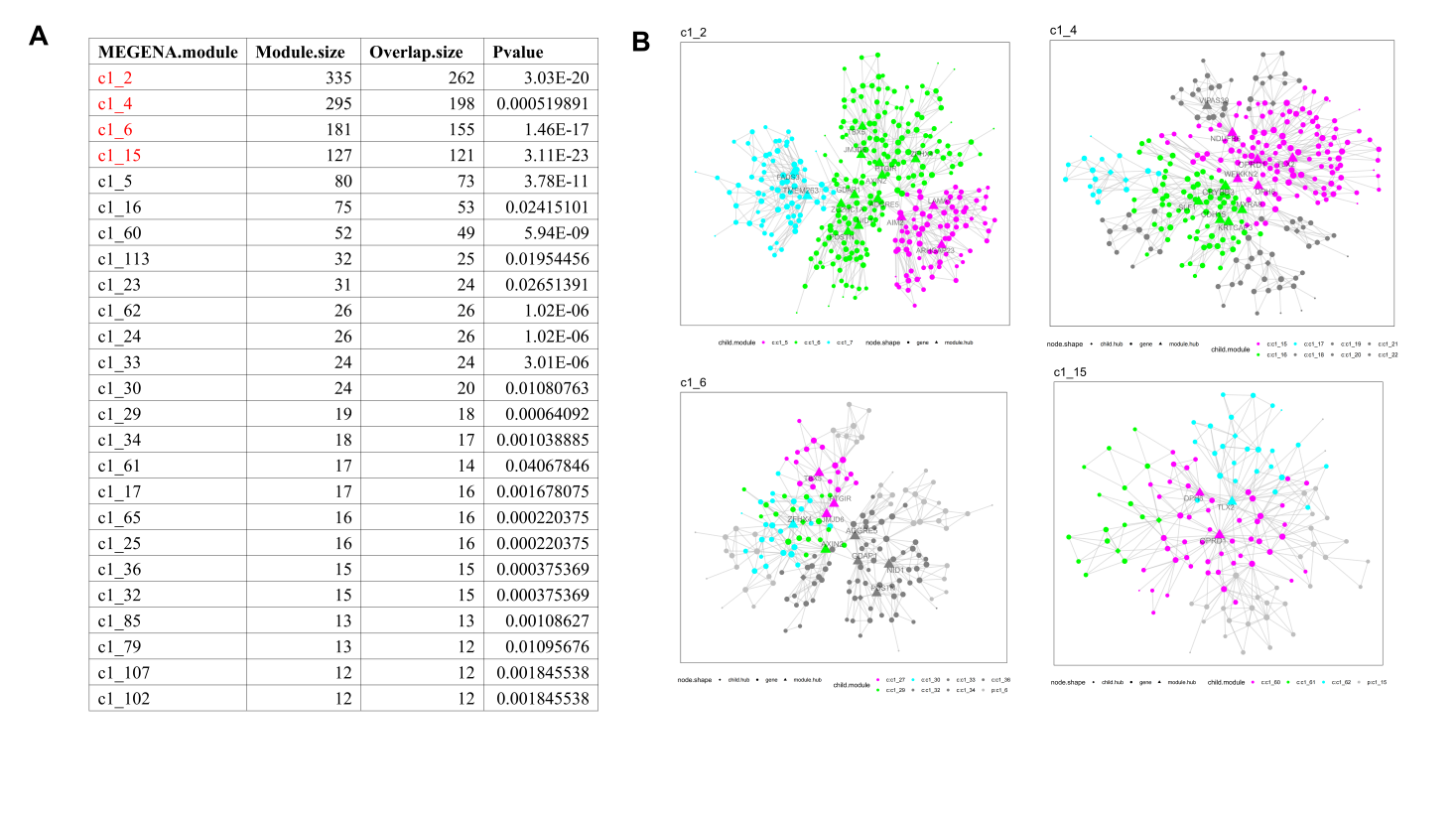
**

**Figure S1. MEGENA hypergeometric distribution test. (A)** The MEGENA module genes were hypergeometric distribution tested with the WGCNA turquoise module genes. (B) The MEGENA network showing the top 4 modules. Each color represents one module, and triangles represent key genes in the module.

**
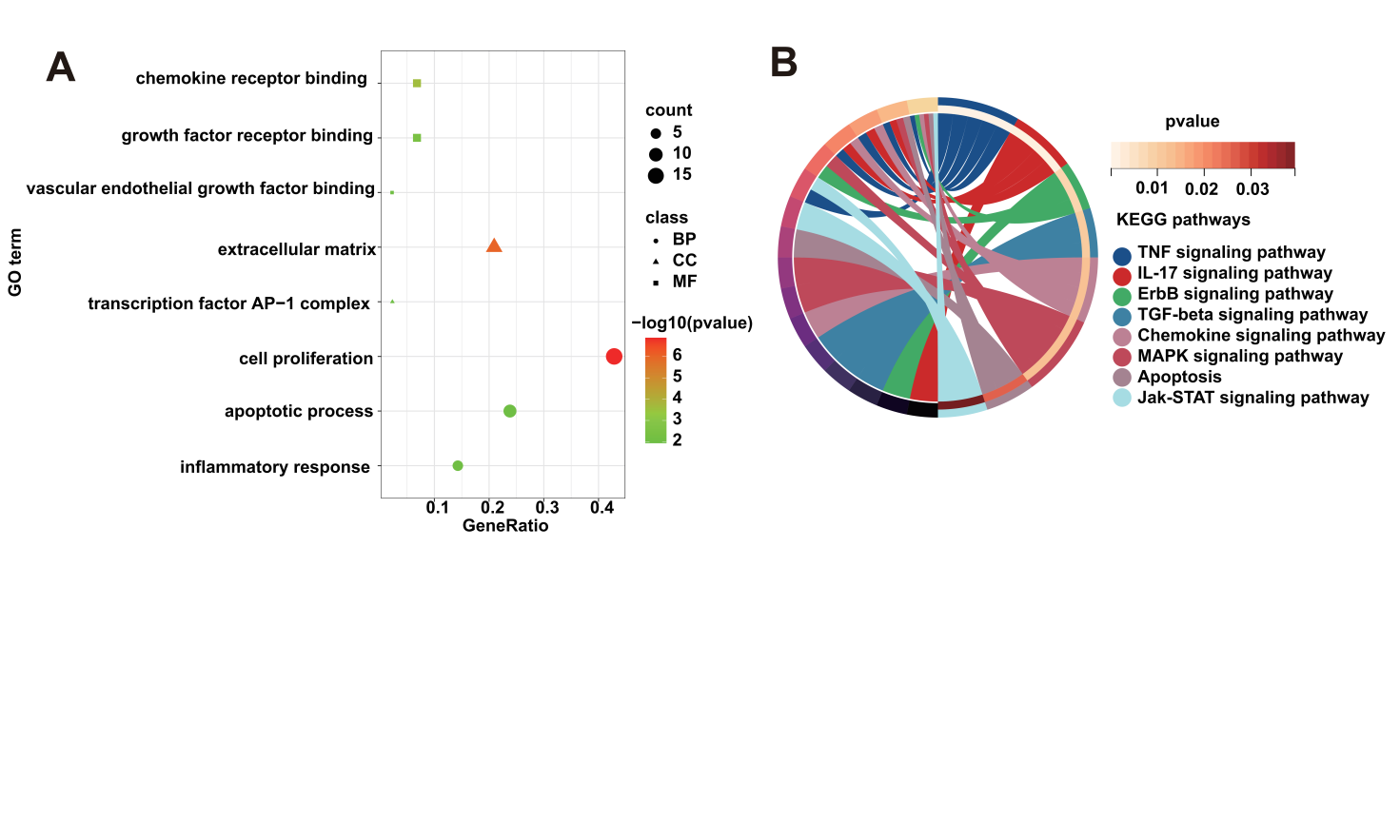
**

**Figure S2. GO and KEGG analysis of the** **44 keloid/immune-related DEGs.** **(A)** GO enrichment analysis of keloid/immune-related DEGs. **(B)** KEGG pathway enrichment analysis of keloid/immune-related DEGs.


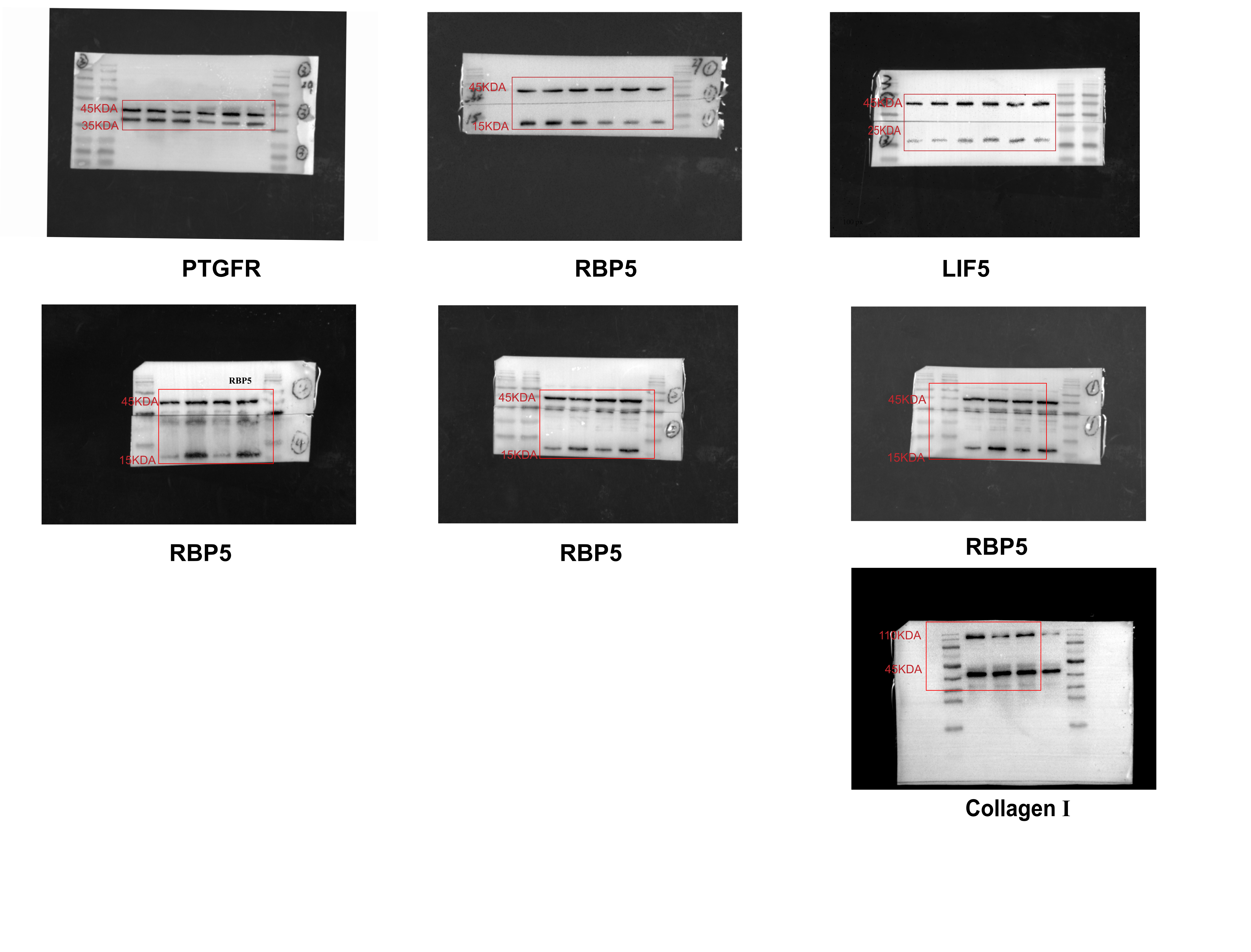


**Figure S3. Image of the western blot in this study.**
